# P-1648. Impact of Provider Education Versus Prospective Audit and Feedback on Outpatient Antibiotic Durations for Endodontic Infections at an Urban Veteran’s Affairs Medical Center

**DOI:** 10.1093/ofid/ofae631.1814

**Published:** 2025-01-29

**Authors:** Ryan Kuhn, Sorabh Dhar, Suganthini Krishnan Natesan

**Affiliations:** John D Dingell VA Medical Center, Detroit, Michigan; Wayne State University/Detroit Medical Center, John Dingell VAMC, Detroit, Michigan; John D. Dingell VA Medical Center, Wayne State University, OAKLAND TOWNSHIP, Michigan

## Abstract

**Background:**

Despite evidence-based practice guidelines published by the American Dental Association, data indicates that antibiotic prescribing by dentists is often inappropriate. Optimal antimicrobial stewardship (AS) strategies to ensure appropriate antibiotic durations of therapy for endodontic infections are not well defined.

**Methods:**

This single center, retrospective review evaluated the impact of provider education alone versus pharmacist-directed prospective audit and feedback (PAF) on outpatient antibiotic durations for endodontic infections. For the first phase of the intervention, staff dentists were educated regarding indications for antibiotic therapy and the recommended duration of therapy (3-7 days) for treatment of endodontic infections. For the second phase of the intervention, the Clinical Pharmacist used real-time electronic medical record alerts to perform PAF of antibiotic prescriptions for endodontic infections. The mean duration of antibiotic therapy and the number of antibiotic courses with a duration of 10 days or longer were collected. Data from the pre-intervention period (12/1/2018 to 11/31/2019), post-intervention period 1 (1/1/2022 to 12/31/2022) and post-intervention period 2 (4/1/2023 to 12/31/2023) were compared.

**Results:**

Compared to the pre-intervention period, the mean duration of antibiotic therapy decreased from 7.8 days to 7.4 days and the number of treatment courses of 10 days or longer decreased by 40% (pre-intervention period, 22.5% [64/284]; post-intervention period 1,13.5% [52/384]; *P* =.003) in post-intervention period 1. Compared to post-intervention period 1, the mean duration of antibiotic therapy decreased from 7.4 days to 6.5 days and the number of treatment courses of 10 days or longer decreased by 84% (post-intervention period 1,13.5% [52/384]; post-intervention period 2, 2.2% [4/182]; *P* < .001) in post-intervention period 2.

**Conclusion:**

While provider education and pharmacist-directed PAF were both effective strategies to decrease inappropriate antibiotic durations of therapy for endodontic infections, PAF was the optimal AS strategy in this setting. Clinical Pharmacists play an essential role in outpatient AS programs and should be engaged with dental clinics to help to improve antibiotic prescribing.

**Disclosures:**

**All Authors**: No reported disclosures

